# Immune Response after Skin Delivery of a Recombinant Heat-Labile Enterotoxin B Subunit of Enterotoxigenic *Escherichia coli* in Mice

**DOI:** 10.3390/pharmaceutics14020239

**Published:** 2022-01-20

**Authors:** Melibea Berzosa, Alzbeta Nemeskalova, Amaia Zúñiga-Ripa, Miriam Salvador-Bescós, Eneko Larrañeta, Ryan F. Donnelly, Carlos Gamazo, Juan M. Irache

**Affiliations:** 1 Department of Microbiology and Parasitology, Institute of Tropical Health, IDISNA, University of Navarra, 31008 Pamplona, Spain; mberzosa.1@alumni.unav.es (M.B.); Alzbeta.Nemeskalova@vscht.cz (A.N.); azuniga@unav.es (A.Z.-R.); msalvadorb@unav.es (M.S.-B.); 2 Department of Analytical Chemistry, University of Chemistry and Technology Prague, Technická 5, 166 28 Prague, Czech Republic; 3 Medical Biology Centre, School of Pharmacy, Queen’s University Belfast, 97 Lisburn Road, Belfast BT9 7BL, UK; e.larraneta@qub.ac.uk (E.L.); r.donnelly@qub.ac.uk (R.F.D.); 4 Department of Pharmacy and Pharmaceutical Technology, University of Navarra, 31008 Pamplona, Spain

**Keywords:** enterotoxigenic *Escherichia coli* (ETEC), intradermal vaccine, LTB subunit, dissolving microneedles

## Abstract

Enterotoxigenic *Escherichia coli* (ETEC) infections have been identified as a major cause of acute diarrhoea in children in developing countries, associated with substantial morbidity and mortality rates. Additionally, ETEC remains the most common cause of acute diarrhea of international travellers to endemic areas. The heat-labile toxin (LT) is a major virulence factor of ETEC, with a significant correlation between the presence of antibodies against LT and protection in infected patients. In the present work, we constructed a recombinant LTB unit (rLTB) and studied the capacity of this toxoid incorporated in microneedles (rLTB-MN) to induce a specific immune response in mice. MN were prepared from aqueous blends of the polymer Gantrez AN^®^ [poly (methyl vinyl ether-*co*-maleic anhydride)], which is not cytotoxic and has been shown to possess immunoadjuvant properties. The mechanical and dissolution properties of rLTB-MNs were evaluated in an in vitro Parafilm M^®^ model and in mice and pig skin ex vivo models. The needle insertion ranged between 378 µm and 504 µm in Parafilm layers, and MNs fully dissolved within 15 min of application inside porcine skin. Moreover, female and male BALB/c mice were immunized through ear skin with one single dose of 5 μg·rLTB in MNs, eliciting significant fecal anti-LT IgA antibodies, higher in female than in male mice. Moreover, we observed an enhanced production of IL-17A by spleen cells in the immunized female mice, indicating a mucosal non-inflammatory and neutralizing mediated response. Further experiments will now be required to validate the protective capacity of this new rLTB-MN formulation against this deadly non-vaccine-preventable disease.

## 1. Introduction

Enterotoxigenic *Escherichia coli* (ETEC) strains cause severe diarrheal illness and are a leading cause of death among children under five in low incoming countries [1]. Furthermore, ETEC strains are the leading cause of traveller’s diarrhea [2]. Consequently, there is a critical need for a vaccine against this pathotype [1]. However, the development of an effective vaccine against ETEC poses a great challenge, due to its pronounced genetic and antigenic variability [3]. Following ingestion, once the bacteria colonizes the small intestine via adhesins, ETEC produces enterotoxins that lead to diarrhea. Virulent ETEC strains mainly produce heat-labile toxin (LT), which causes the release of electrolytes and the secretion of water, resulting in the typical watery diarrhea [4,5,6]. ETEC-infected patients present LT-antibodies in intestinal lavage fluid, breast milk, and saliva, and a significant correlation was found with protection [7,8,9,10]. Thus, some promising data are available regarding the use of LT exotoxins in vaccination.

The skin has been considered in this work as a route for vaccination to avoid stomach and intestinal physicochemical challenges and, at the same time, to get the benefit of the connection among mucosal and skin-associated lymphoid tissues where dendritic cells (DC) are going to take up the vaccine, process it, transport it, and present it to T lymphocytes in the draining lymphoid organs [11] LT is composed of the toxic A subunit and the non-toxic pentameric B subunit (LTB) that bind to the ganglioside GM1, which acts as receptor port of cellular entry. In the present work, we constructed a recombinant LTB unit (rLTB) and studied the capacity of this protein loaded into dissolving microneedle (MN) arrays to induce a specific immune response in mice. To do this, the rLTB was embedded in Gantrez AN^®^ in a form of dissolving MNs patches (rLTB-MN). Antigen-containing MNs consist of micrometric projections that are able to facilitate the entry of the antigen through the skin. Gantrez AN^®^ was selected for its immunoadjuvant properties and low cytotoxicity. We previously reported, for the first time, that this formulation of MNs loaded with outer membrane vesicles of *Shigella flexneri* was immunogenic and protective in a murine model of shigellosis [12].

The aim of this study was then to develop MNs from the Gantrez AN^®^ polymer for transdermal delivery of rTLB. The mechanical strength and dissolving properties of the conformed rLTB-MNs, and the insertion capability in neonatal porcine skin were investigated. Finally, their immunogenic capacity in mice was evaluated, showing a marked bias to Th2 response, with high levels of specific mucosal IgA.

## 2. Materials and Methods

### 2.1. Construction of a Recombinant Plasmid Encoding LTB Subunit Gene

The ETEC10407 *eltB* gene encoding the LTB protein was amplified with the primers: FweltB (5′-AAGGAGATATACATATCGGAATGAATTATGAATAAAG-3′) and RveltB (5′-GGTGGTGGTGCTCGAAGTTTTCCATACTGATTGCC-3′). Then, it was cloned into the NdeI and XhoI sites of the Pet-21a(+) vector to generate pET21-*eltB*. The integrity of the plasmid was corroborated by PCR and sequencing [Applied Medical Research Centre (CIMA) of the University of Navarra, Pamplona, Spain].

### 2.2. Expression of rLTB Recombinant Protein

#### 2.2.1. Proteomic Analysis

The recombinant histidine-tagged pET21-eltB expression plasmid was transformed to *E. coli* Stellar (Clontech) and further in *E. coli* BL21 (DE3) for recombinant protein rLTB expression. A single colony of *E. coli* BL21 transformed with pET21-*eltB* plasmid was inoculated into Luria-Bertani (LB) medium supplemented with ampicillin (100 µg/mL), followed by agitation at 120 rpm at 37 °C to reach an O.D._600nm_ of 0.6. Then, an inoculum was transferred to a 500 mL fresh LB medium and incubated at 37 °C with 120 rpm agitation overnight. Induction of the *eltB* gene expression was performed with the addition of 0.3 mL 1 M isopropyl β-d-1-thiogalactopyranoside (IPTG) at 16 °C. After 12 h incubation, the cellular pellet was collected (6000× *g*, 4 °C, 20 min) and resuspended in ice-cold lysis buffer containing protease inhibitors (100 mM PBS, pH 7.5, 200 mM NaCl, 20% glycerol, protease inhibitor, Roche) and lysed with a high-pressure French Press (SIM AMINCO) at 14.000 psi; twice. The supernatant was collected following centrifugation at 20,000× *g* for 20 min.

#### 2.2.2. Purification of rLTB Recombinant Protein

A sample of 8 mL of supernatant was homogenized in 1 mL nickel-nitrilotriacetic acid (Ni-NTA) agarose (Qiagen, Darmstadt, Germany) at 4 °C, 1 h, 200 rpm, and filled in a column (Qiagen, Darmstadt, Germany) pre-equilibrated with 20 mM lysis buffer. The column was washed four times with imidazole buffer (50 mM NaH_2_PO_4_, 300 mM NaCl, 20 mM imidazole; pH 8) and finally, the column was eluted with the same equilibration buffer. The purified recombinant proteins were collected and analyzed by SDS-PAGE stained with Coomassie blue R-250. Different concentrations of the cholera toxin (CT, Sigma, St. Louis, MO, USA) were used as a reference pattern. After electrophoresis, the proteins were transferred onto nitrocellulose membranes (Whatman Protran^®^; pore size of 0.45 µm) and incubated in blocking buffer overnight at 4 °C (10 mM Tris, pH 7.5, 0.9% NaCl, 3% BSA). The rLTB protein was detected by incubation of the membrane with an anti-LT (A + B) antibody (ab188541, Abcam, Cambridge, UK) for 4 h. Finally, the membrane was incubated with peroxidase-conjugated goat anti-rabbit antibody and the bands were developed by incubation with a substrate/chromogen solution (H_2_O_2_/4-chloro-1-naphthol). The signal of the resulting bands was quantified by densitometry and rLTB concentration was determined by extrapolation of its signal in the reference pattern data.

#### 2.2.3. Analysis of rLTB Recombinant Protein Conformation

To investigate if the rLTB recombinant protein once purified presented in a native state, ELISA and dot-blot techniques were used. ELISA assay was performed in 96-well plates (MaxiSorb; Nunc, Wiesbaden, Germany), coated with 100 μL of 0.5 µg/mL GM1 ganglioside in PBS at room temperature overnight. The plates were washed four times with TBS-Tween 0.05% and nonspecific binding sites were blocked with 3% BSA in PBS for 1 h at room temperature. After that, the plates were washed again, and rLTB or CT (Sigma, St. Louis, MO, USA) diluted in PBS were added. After incubation at 37 °C for 2 h, bound proteins were detected by using a rabbit anti-LT antibody (1: 5000 in PBS), followed by an anti-rabbit alkaline phosphatase-conjugated secondary antibody (1:1000 in PBS). Finally, absorbance at 405 nm was recorded. The mean values from three independent experiments were reported.

Additionally, a dot-blot assay was carried out with the following conditions: two μL of each sample was fixed on a nitrocellulose filter using a Bio-Dot microfiltration apparatus (Bio-Rad, Hercules, California, CA, USA). The filter was blocked overnight with 3% BSA in PBS and incubated for 4 h with an anti-LT antibody diluted 1:25 or 1:100 in PBS. Bound antibodies were detected with peroxidase-conjugated goat anti-rabbit IgG (Nordic) 1:1000 for 1 h and antibody reactivity was visualized using H_2_O_2_/4-chloro-1-naphthol.

### 2.3. Polymeric MN Arrays Formulation

**Figure 1:**
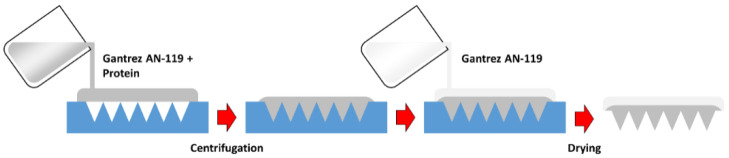
Diagram showing microneedle preparation. Formulation containing rLTB protein was poured into each silicon micromold. The polymer used for this work was Gantrez^®^ AN-119.

MN arrays were prepared using poly(methyl-vinyl-ether-*co*-maleic anhydride) (Gantrez^®^ AN-119) (Mw = 200 kDa), whose physicochemical properties were characterized before [13] The procedure is illustrated in Figure 1. To prepare the MN arrays, 50 mg of a solution containing 30% (*w*/*v*) Gantrez AN-119 and 5 μg of the rLTB protein was poured into silicone micromolds. Each mold, 0.7 cm × 0.7 cm, contained 361 (19 × 19) conical-shaped micro-needles. Needle height was 500 μm and the width of each needle at the base was 300 μm. Subsequently, the molds were centrifuged at 2850× *g* for 15 min to fill the mold cavities. Finally, 50 mg of a 30% (*w*/*v*) Gantrez solution in deionized water was added to prepare a thicker baseplate containing no protein. Finally, MNs were dried at room temperature (RT) for 24 h and removed from the molds.

### 2.4. Mechanical Characterization of MNs

#### 2.4.1. Insertion Test

MN insertion was evaluated using a skin simulant based on Parafilm M^®^ [14]. For this purpose, eight layers of Parafilm were stacked together. Subsequently, MN patches were inserted into the skin model using a TA.XTPlus Texture Analyser (Stable Micro Systems, Surrey, UK). A force of 32 N was applied for 30 s using a speed of 1.19 mm/s. These insertion conditions simulate manual insertion by human volunteers [14]. Finally, the insertion profile was calculated by evaluating the percentage of holes in each layer of Parafilm. A Leica EZ4 Dc (Leica, Wetzlar, Germany) digital microscope equipped with two polarizer filters was used to measure the number of holes in each Parafilm layer. The insertion profile was calculated considering that each Parafilm layer has a thickness of 126 µm [14].

#### 2.4.2. Skin Insertion Studies

Ex vivo dissolution studies were performed using full-thickness neonatal porcine skin, obtained from stillborn piglets, and used immediately. Neonatal porcine skin has been previously used as a human skin model [15,16]. MN were inserted manually following the protocol previously reported [17]. Subsequently, an EX1301 optical coherence tomography (OCT) microscope (Michelson Diagnostics Ltd., Kent, UK) was used to evaluate MN dissolution over a period of 1 h.

### 2.5. Immunization Studies

Animals were treated according to ethical standards (CEEA, Ethical Committee for Animal Experimentation at the University of Navarra, Spain; approval code: 027/20, 22 May 2020). In order to determine the suitability of rLTB-MN, eight-week-old female and male BALB/c mice (20 ± 1 g) (Envigo, Indianapolis, IN, USA) were separated into randomized groups of five animals and immunized with rLTB (5 μg) through the intradermal route or using MNs. These MNs were manually applied on the dorsal surface of the ear of the mouse. After applying pressure for 1 min, they were attached by an adhesive band and removed after 24 h. Non-immunized mice were used as controls.

### 2.6. Detection of Fecal Antibodies by ELISA

Fecal pellets were taken before immunization (time 0) and at weeks 1, 2, 3, and 4 post-immunization. The collected and weighed feces were adjusted to 1 mg/mL with PBS containing protease inhibitor cocktail (Sigma-Aldrich, Cambridge, UK). Debris was cleared by centrifugation at 10,000× *g* for 10 min, and the supernatants were collected and stored at −20 °C until use. A GM_1_-ELISA was used to determine LTB-specific fecal IgA. Briefly, 96-well plates (MaxiSorp, Nunc, Wiesbaden, Germany) were coated with 100 μL of 0.5 µg/mL GM_1_ in PBS at room temperature overnight. The plates were washed four times with TBS-Tween, and nonspecific binding sites were blocked by 3% BSA in PBS for 1 h at room temperature. After washing, rTLB or the commercially available cholera toxin CT (Sigma-Aldrich, Saint Louis, MO, USA) diluted in PBS were added and incubated at room temperature for 4 h. Bound proteins were detected by using anti-LT·IgG antibodies, followed by alkaline phosphatase-conjugated secondary IgA antibodies. ABTS-H_2_O_2_ was used as chromogenic-substrate and absorbance at 405 nm was recorded.

### 2.7. Determination of IL-17A, TNFα IFN-γ, and IL-4 Production

In order to determine the pattern of cytokines elicited after immunization, naive and immunized mice were sacrificed by cervical dislocation on day 28 after immunization. Spleens were removed and placed in RPMI 1640 medium (Gibco-BRL, Bleiswijk, The Netherlands). Splenocytes, 4 × 10^5^ cells in RPMI 1640 medium supplemented with 1 IU/mL penicillin, 1 µg/mL streptomycin, and 10% fetal bovine serum (Gibco-BRL, Grand Island, New York, NY, USA) were incubated with CT (1 µg/mL) in a final volume of 200 µL per well. PMA/Ionomicine (100 ng/mL and 4 µg/mL) treated cells were used as positive controls The culture supernatants were collected for cytokine assay at 72 h after the stimulation and were kept frozen at −80 °C. Cytokines were quantified by cytometry (Acoustic Focusing Cytometer Attune^®^, Applied Biosystems, Foster City, CA, USA) using a bead CBA mouse Th1/ Th2/ Th17 kit (Biolegend, San Diego, CA, USA).

### 2.8. Statistical Analysis

ANOVA tests were used to analyze the significance of the differences between the experimental and the control groups.

## 3. Results

### 3.1. rLTB Construction and Expression

In order to use the LT toxin as an immunogen, one approach to overcome and reduce its inherent toxicity is the use of the LTB non-toxic subunit. In this work, the *eltB* gene coding LTB was cloned into a pET21 vector for expression of rLTB in *E. coli* BL21 (DE3).

**Figure 2:**
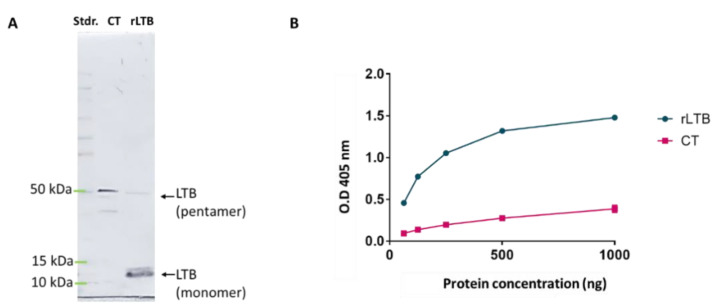
Biological reactivity of rLTB analyzed by immunoblotting (**A**) and a GM1 ganglioside-binding assay (**B**). Immunoblotting was performed using specific sera against LT, using the cholera toxin (CT) as a control; the identity of reacting bands is indicated. Panel B shows the degree of binding affinity of rLTB for the specific receptor GM1. The ELISA experiment was performed by incubation of coated GM1 with purified rLTB or with CT-B as a positive control.

After induction with IPTG, the rLTB product obtained after bacterial lysis was purified by affinity chromatography and fractions were analyzed by SDS-PAGE and immunoblotting. A band of the expected molecular mass for monomeric LTB (12 kDa) was detected to react with anti-histidine antibodies (Figure 2), suggesting that the protein was expressed. The obtained concentration of the purified rLTB was 0.2 mg/mL.

The biological reactivity of rLTB was analyzed by a GM1 ganglioside-binding assay. Increasing concentrations of either rTLB or the commercially available cholera toxin B subunit (CTB) were tested. As shown in Figure 2, rLTB bound efficiently to GM_1_ ganglioside, suggesting that plasmid-produced LTB assembles into a native state. In this assay, the maximum levels of binding detected with commercial rLTB were observed at a concentration of 500 ng/mL, and this degree of binding affinity was much higher than that associated with CTB (Figure 2).

### 3.2. Polymeric MN Arrays Formulation Containing rLTB

Once the rLTB protein was obtained, MNs containing the recombinant toxoid (5 µg of the protein) were developed. Additionally, blank MNs were prepared as a control using Gantrez AN-119^®^ only. These MN arrays were prepared successfully. Blank MN arrays and protein-loaded arrays presented an equivalent appearance.

### 3.3. Skin Insertion and Dissolution Studies

**Figure 3:**
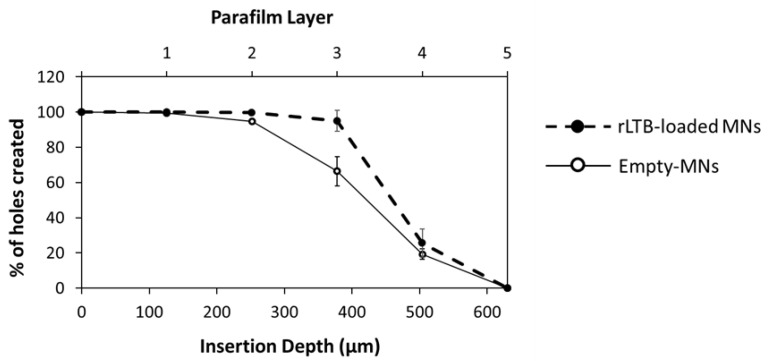
Percentage (%) of holes created by empty or rLTB-loaded Gantrez^®^ AN119 MN patches (MNs) in different Parafilm layers. Error bars represent S.D.

Blank and protein-loaded MN arrays showed similar insertion profiles in Parafilm M^®^ (Figure 3). Both types of MN arrays were successfully inserted through the first three layers of Parafilm. It was noticeable that the number of holes created in the third Parafilm layer was lower for blank MN arrays. However, this test was designed to establish a range for MN insertion. For the MN formulations evaluated in this study, total needle insertion ranged between the third (378 µm) and the fourth (504 µm) Parafilm layer. These results are superior to conical 19 × 19 MN arrays with similar dimensions that under similar insertion conditions were capable of insertion between the second and the third layer of the Parafilm skin simulant [14].

### 3.4. Insertion in Neonatal Porcine Skin

**Figure 4:**
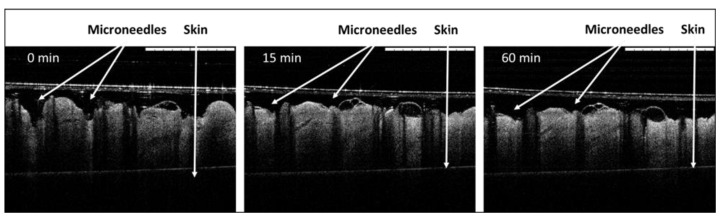
Optical coherence tomographic images of dissolving rLTB-loaded Gantrez^®^ AN-119 MNs in porcine skin. Images show in vitro dissolution at times 0, 15, and 60 min after insertion into porcine skin. White arrows indicate MNs or skin. Scale bars: 1 mm.

Ex vivo insertion studies were performed in excised neonatal porcine skin, as it is a good model for human skin [17]. MN insertion and dissolution were evaluated using optical coherence tomography. MNs prepared using Gantrez AN-119 were dissolving as reported previously by Pastor et al. [12]. Figure 4 shows MN dissolution inside porcine skin, which indicates that MNs fully dissolved within 15 min of application. These results are in line with previously-reported MN arrays prepared using the same polymer.

### 3.5. Evaluation of Mucosal Antibody Responses

**Figure 5:**
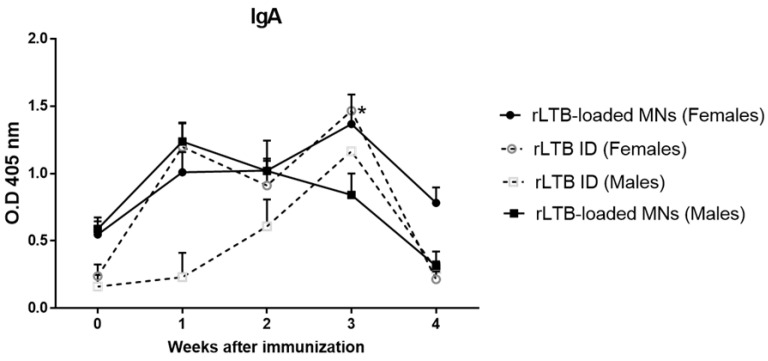
rLTB-specific IgA antibodies in immunized mice. BALB/c mice (females and males) (*n* = 5) were immunized by intradermal (ID) or MN arrays with 5 μg of recombinant of heat-labile enterotoxin B (rLTB) from Enterotoxigenic *Escherichia coli* (ETEC) H10407 (ATCC 35401). Specific IgA antibodies against rLTB in mice serum were determined by GM1-ELISA (* *p* < 0.05 vs. pre-immunization time). Error bars represent S.D.

IgA antibodies at the intestinal lumen are the major line of defense against exotoxins. Therefore, we first investigated the presence of fecal antibodies following MN or intradermal administration of free rLTB. Results indicate that immunization with 5 μg of rLTB embedded into MNs elicits a significant mucosal IgA response (Figure 5).

### 3.6. Analysis of Cell-Mediated Immune Responses

To assess the effect of rLTB-MN on the generation of a cell-mediated immune response in mice, spleen cells were isolated and in vitro and re-stimulated with CT.

**Figure 6:**
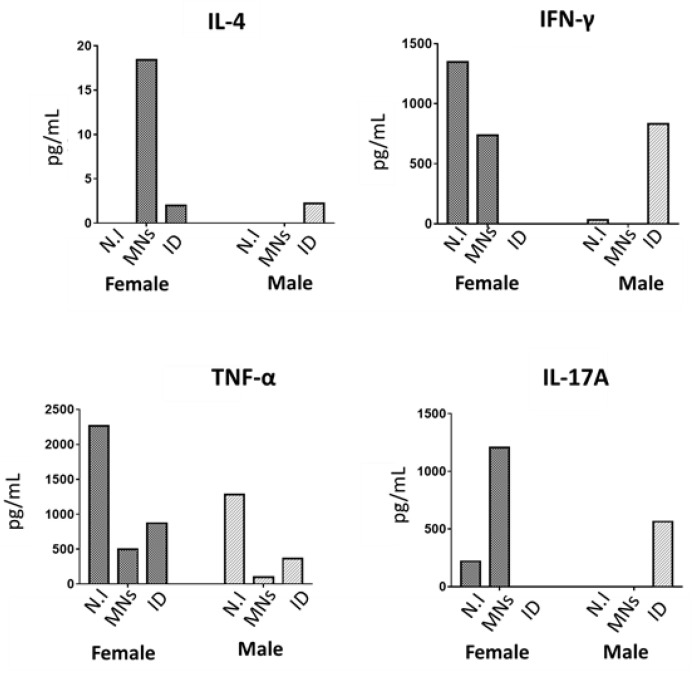
Splenic cytokine profile in immunized BALB/c female and male mice with rLTB-loaded microneedle patches (MNs) or with rLTB through intradermal route (ID). Non-immunized (N.I) mice were used as a negative control. Cytokine levels were determined by ELISA and expressed in pg/Ml.

Results indicated that female mice immunized with rLTB-MN showed higher levels of IL-4, IFN-γ, and IL-17 compared to both male mice immunized with rLTB-MN and mice immunized by intradermal route (Figure 6).

## 4. Discussion

Most ETEC strains isolated from symptomatic infected patients produce the LT enterotoxin that accounts for the bulk of diarrheal disease burden [5,6].

LT is a major virulence factor of ETEC, not only because it induces the associated diarrheal disease, but because it also enhances bacterial adherence to the enterocytes. Thus, in the first instance, the non-toxic B LTB structure interacts with the cellular surface of GM1 ganglioside and, after that, the toxic LTA subunit is internalized inducing a massive secretion of electrolytes and water into the lumen. Consequently, LTB is considered as the main target to be neutralized by the host immune system and, consequently, to be the main component in vaccines against ETEC infections. In fact, there is a significant correlation between the presence of antibodies against LT and the induced protection, and Etvax, the most advanced vaccine candidate, demonstrated good immunogenicity with strong serum IgA and IgG responses to LT exotoxin [3]. Following this rationale, in this current work, we have successfully obtained an affordable recombinant construct of the non-toxic LTB subunit in its native state conformation capable to interact efficiently with GM1. The second challenge was to use the correct delivery system and route for its safe administration to induce a Th2 mediated response capable to produce mucosal neutralizing IgA. Secretory IgA specific against LT is widely thought to be important in protection [18,19]. This response was associated with the development of high avidity antibodies suggesting that they may participate in neutralizing responses [20,21]. In addition, recent studies have shown that IgA nanobodies specific against LTB have been proven efficient for mucosal protection against ETEC in piglets [22,23]. The skin is a notable route of vaccine delivery, capable of eliciting both mucosal and systemic immune responses [24]. This route of administration is attractive for the rapid and wide biodistribution of the antigens for painless self-administration, and for the possibility of avoiding the use of needles, which would circumvent problems related to needle-phobia, needle-associated infections and would not require healthcare personnel [25]. Among the different strategies to deliver the vaccine components through the skin *stratum corneum*, we used here dissolving MNs based on Gantrez^®^. This polymer has been extensively employed for pharmaceutical applications, due to its mucoadhesive properties and low toxicity. Specifically, we used Gantrez^®^ AN-119, as we have previously shown that this particular polymer is non-cytotoxic and has immunoadjuvant properties [26,27]. In this work, rLTB loaded MNs were successfully produced and showed excellent insertion properties when analyzed in both Parafilm M^®^ and porcine skin. Moreover, OCT showed that the MNs could successfully penetrate porcine skin and the microprojections of the patches containing the protein were dissolved after application. Finally, the patches were applied into murine ears, where they did not show any significant irritation or necrotic signs 24 h post insertion.

The insertion profiles combined with the fast dissolution kinetics make these types of MN arrays ideal for vaccine delivery applications. These patches will not require long wearing times to deliver their cargo. Additionally, 15 min after applications, needle tips will be completely dissolved so, when the patches are removed, they cannot be reinserted into anybody else. Therefore, this system prevents reinfection and the need for sharp waste disposal [28]. These aspects are especially important when developing MN-based vaccine delivery systems for developing countries. Gantrez AN-119 was used previously to prepare MN arrays for intradermal vaccination against Shigellosis [12]. The dissolution time of the MN tips described in this work was consistent with the findings reported here. MN tips dissolved within 1 h. However, the dissolution time of the MN tips described here is slightly faster (15 min vs. 45 min). This is due to the difference in needle geometry that facilitates a deeper insertion for the arrays described in this work (three vs. two layers in the Parafilm test). In addition to the insertion and dissolution properties of this polymer, it is important to note that, the use of aqueous gels for the development of dissolving MN arrays has been extensively reported. This is a clear advantage for scale-up manufacturing. Similar aqueous gels such as hyaluronic acid have been used before to develop MN commercial products [29] Accordingly, the scale-up manufacturing of this type of MN system is technically feasible.

The insertion properties of the MN arrays were corroborated in the mice immunization studies, which confirmed that the recombinant protein embedded in MNs elicit marked Th2 immunity with high levels of IgA in feces from week one after immunization. Remarkably, we found sex differences in these immunogenic studies. The sexual dimorphisms in the skin immune processes have been broadly investigated at a cellular and molecular level [30,31,32,33]. For instance, it has been described that the density of the Langerhans cells (LC) in the skin-ears of female mice is significantly higher than in males. LCs are antigen-presenting cells mainly committed to contributing to maintaining a tolerance status based on Th2/TH17 polarization characterized by their IL10 and IL17A production [34,35,36]. Our results indicate that the levels of IL-17A were higher in female than in the male animals that had received loaded MNs. It is known that TLR2-activated LCs promote Th17 polarization, and, in fact, the LTB subunit is able to interact with TLR2, supporting this possibility [37,38]. In addition to LCs, there are other epidermal players involved in the general trend toward a Th2-anti-inflammatory state in the skin. Thus, keratinocytes (KCs) cells express immunoregulatory and Th2 cytokines, such as TNF-α that induces the expression of IL-4 in DCs, which promotes a Th2 response [39]. Our results showed both higher levels of TNF-α but mainly of IL-4 in the MN-immunized female mice. Finally, another important cellular component of the epidermal immune system is *γδ* T cells [40]. These lymphocytes interact extensively with KCs and are able to recognize unprocessed antigens such as bacterial toxins, including the cholera toxin closely related to the LT enterotoxin [41,42]. *γδ* T cells also express TLR2 and could also be involved in the over-production of IL-17A by these cells [43]. Furthermore, IL-17A released from *γδ* T cells promote KC activation and the recruitment of key effector immune cells to the skin. As a consequence, and in line with our own results, activation of *γδ* T cells by LTB mediated by TLR-2 will condition the predominant Th2 observed with increased IgA levels in feces. IL-17A plays a critical role in maintaining mucosal integrity, including the regulation of intestinal IgA production [44] and it has been clearly shown that Th2-IL17 elicits production of secretory IgA at mucosal surfaces to provide protection against ETEC infections [45].

Importantly, LT has been shown to induce IL-17A and IFN-γ [5,46]. Considering that in controlled ETEC human infection studies, IL-17A and IFN-γ increase significantly in subjects with moderate-severe diarrheal illness [47], and high levels of IL-17A are elicited after rLTB-MN immunization, our results encourage additional in vivo studies using this particular delivery system.

On the other hand, in the current work, we included a control of intradermal (i.d.) rLTB administration, showing the inability to produce the above-described immune response. These differences, with respect to MN delivery, likely reflect distinct pathways of immunological priming. We hypothesize that the unique environment of the intestinal epithelium may explain the distinct effects of LT on the epidermis (MN) versus dermis administration (i.d.). Thus, LTB interaction with KCs and LCs via TLR-2 (MN immunization) promotes the polarization of naïve Th toward Th2 cells. In contrast, LTB interaction with dermal DCs (ID immunization) induces differentiation into a distinct population with regulatory immunosuppressive effects [48].

Insights from this work may provide new designs for skin vaccination strategies. In particular, and considering the potent immunoadjuvant properties of the LTB subunit [49], the MN formulation described here is suitable to be co-loaded with ETEC antigenic complexes to get a broad-spectrum ETEC vaccine. This skin delivery system is a simple, affordable, and easy-to-use way of antigen administration, facilitating the immunization processes in low-income countries, following the WHO guidelines for the development of vaccines against this worldwide cause of diarrheal disease [50]. Before this technology can be applied clinically, MN manufacturing technology should be scaled up. The technology available for MN manufacturing at a large scale is available. Currently, pharmaceutical companies are working actively to develop MN products at an industrial scale [51]. These recent developments are showing that the development of MN-based vaccine products at an industrial scale is feasible.
